# Comparison of Effects of Sodium Chloride and Potassium Chloride on Spray Drying and Redispersion of Cellulose Nanofibrils Suspension

**DOI:** 10.3390/nano11020439

**Published:** 2021-02-09

**Authors:** Guihua Yang, Guangrui Ma, Ming He, Xingxiang Ji, Weidong Li, Hye Jung Youn, Hak Lae Lee, Jiachuan Chen

**Affiliations:** 1 State Key Laboratory of Biobased Material and Green Papermaking, Qilu University of Technology, Shandong Academy of Sciences, Jinan 250353, China; ygh@qlu.edu.cn (G.Y.); mgr12s@163.com (G.M.); xxjt78@163.com (X.J.); 17854116982@163.com (W.L.); page94@snu.ac.kr (H.J.Y.); lhakl@snu.ac.kr (H.L.L.); 2 State Key Laboratory of Pulp and Paper Engineering, South China University of Technology, Guangzhou 510640, China; 3 Department of Agriculture, Forestry and Bioresources, Research Institute of Agriculture and Life Sciences, College of Agriculture and Life Sciences, Seoul National University, 1 Gwanak-ro, Gwanak-gu, Seoul 08826, Korea

**Keywords:** potassium chloride, sodium chloride, cellulose nanofibrils, dpray drying, redispersion

## Abstract

Cellulose nanofibrils (CNFs) were exposed to the same levels of potassium chloride (KCl) and sodium chloride (NaCl) before being subjected to spray drying. The effect of NaCl and KCl on the size of atomized droplets and the hydrogen bond retardation between CNFs was investigated by characterizing product morphology, particle size distribution, dispersion stability in aqueous system, and surface chemistry. The results showed that the CNF suspensions treated with KCl could be atomized into smaller droplets during spray drying, and then CNF powder with smaller sizes could be obtained. As the agglomeration was less, and the CNF with KCl addition had good dispersion stability after redispersion compared with CNF treated by NaCl. Therefore, KCl treatment was an effective method to reduce the agglomeration of CNF during spray drying.

## 1. Introduction

The applications of cellulose nanofibrils (CNFs) as reinforcing components in polymer composites have received widespread attention [1,2,3,4]. Deconstruction of pulp fibers in nanoscale gives CNFs having remarkable mechanical properties with a modulus of elasticity from approximately 10 to 70 GPa or higher (145 GPa) [5]. Such impressive mechanical properties of CNFs can contribute to significant improvement in the mechanical properties of cellulose nanofibrillar-reinforced polymer composites. Biodegradability, low density, worldwide availability and the modifiable surface properties of CNFs offer potential opportunities to develop a new generation of composite materials based on cellulose fibers [6,7]. CNFs are usually prepared and processed in the aqueous environment to take advantage of their hydrophilic nature. Due to the high aspect ratios of CNF, the consistency of CNF suspension is too low to use for many applications. Low consistency of CNF also increases the transportation and storage cost for industrial use. Thus, it is generally believed that there is a need to develop a robust method for drying CNFs, which allows us to reduce the high transportation costs of aqueous suspensions and maintain the nanoscale dimensions of CNF for applications that require dry-form CNFs [8]. Especially, drying of the aqueous suspension of CNFs is essential for their use in the polymer nanocomposites. CNFs, however, tend to agglomerate during drying and other processing steps [6], and this problem causes difficulties in obtaining dried CNFs in a small particulate form.

Spray drying (SD) is a well-established technique that has been used in many areas, including food, pharmaceutical, ceramic, polymer, and chemical industries [9]. The critical cost analysis of the spray-drying process has shown that it has advantages in labor and maintenance costs, which encouraged the acceptance of spray-drying as a standard industrial dehydration method [10,11]. However, during spray drying that requires a direct contact with turbulent hot air for atomization, the particle size of dried CNFs tends to be non-uniform. In particular, large size particles that are not easy to disperse tend to generate in the spray drying process. To solve this problem, approaches of controlling the spray drying process parameters, i.e., feed amount, air intake speed, etc., as methods to control the shape and size of the particles have been made [12,13]. Phenomenological studies showed that the morphologies of spray-dried particles were dependent on the type of materials. Hede et al. reported that the design variables (liquid hole diameter and nozzle angle) and operation variables (liquid feed rate and gas flow rate) of the atomizer could be controlled to produce particles with different morphology [14]. Peng et al. studied the effect of spray drying process parameters such as gas flow rate, liquid feed rate and suspended solid concentration on CNF particle morphology and particle size distribution, and prepared CNF particles with a diameter of 3.95 μm [15]. Spray-drying will be sufficient for the CNF drying method if small CNF agglomerates fall within the end-use criteria for a given application. In general, additives are required to prevent strong hydrogen bonds between CNFs for well redispersion of CNF in water [16], this was because adjusting the drying process parameters could not avoid the irreversible agglomeration of fibers in the drying process [17]. Peng et al. tried to modify CNF in situ with maleic acid [18] in the spray drying process, which reduced the irreversible phenomenon of CNF in the drying process, but it was a modification treatment, which changed the original properties of CNF, and did not detect the redispersibility of CNF after drying. Thus, there was much to be learned on the redispersibility of dried CNFs in aqueous suspensions and design or process parameters in the spray drying process that give small size CNF particles after drying.

It is known that CNF shows a negative charge due to the carboxyl group on hemicellulose [19]. According to Derjaguin–Landau–Verwey–Overbeek (DLVO) theory, the electrostatic interaction between CNFs with carboxyl groups on their surface depends on the types and concentrations of counter ions in the aqueous phase [20]. It has been reported that sodium chloride (NaCl) pretreatment before drying successfully redispersed CNF in an acceptable size range [21,22]. As one type of monovalent alkali metal salt, with similar physical and chemical properties of NaCl, potassium chloride (KCl) has the stronger metallicity than NaCl. To the best of our knowledge, there is little information available in literature about the effect of KCl on CNF redispersion; thus, previous research has failed to considered the differences of KCl and NaCl on the redispersibility of CNF by spray drying.

In the present work, NaCl or KCl solutions were used as dispersing medium for the CNF before spray drying. In order to clarify the effect of NaCl and KCl on inhibiting the hydrogen bonding of CNFs under high temperature spray-drying conditions, sedimentation experiments, and dispersion stability tests were performed along with the particle size measurements. The morphological changes were also analyzed by a high-resolution scanning electron microscope.

## 2. Materials and Methods

### 2.1. Materials

KCl (Mw = 74.55 g/mol) and NaCl (purity ≥ 99.5%, Mw = 58.44 g/mol) were supplied from Komeo Chemical Reagents Co, Ltd., Tianjin, China. Hydrochloric acid (HCl, Mass fraction = 36.0–38.0 w/%) were supplied from Fine Chemical Plant, Tianjin, China, and sodium hydroxide (NaOH, purity ≥ 96.0%, Mw = 40.00 g/mol) were supplied from Da Mao Chemical Reagent Co., Tianjin, China. Dialysis membranes (Spectra/Por3 molecular weight cut off 3500 Da) were purchased from Sigma Aldrich Ltd., San Francisco, CA, USA. Deionized water (Conductivity = 1.0 μS/cm at 25 °C) was used in all experiments. Bleached eucalyptus kraft pulp (Aracruz, Brazil) was used as a raw material for CNF preparation.

### 2.2. CNF Preparation

Raw material fibers at 10.0 wt% was beaten to 100 mL CSF (Canadian standard freeness) using a pulp refiner (PFI-type, (Kumagai, Japan). Then, the beaten pulp fibers at 0.5 wt% were fibrillated through a M-110EH-30 micro fluidizer (Micro fluidics, Newton, MA, USA). Size reduction of the fibers into nanofibrillated cellulose was obtained after 10 passes through 200 μm chambers at a pressure of 200 bar and 15 passes through 200 and 87 μm chambers at a pressure of 1000 bar. The hemicellulose content of CNF prepared in this experiment was 8.9%, and the carboxyl content of CNF measured by conductometric titration was 0.12 mmol/g.

### 2.3. Drying and Redispersion

In order to deprotonate carboxyl groups on CNF, the deprotonation treatment was carried out. The collected CNF suspensions were acidified by adding 0.1 M HCl solution and adjust the pH down to 4.0 [20]. Then, NaOH solution was added to reach a pH of 8, which promoted the deprotonated carboxyl groups. Then a certain amount of NaCl was added to control the ionic strength of CNF suspensions to 10 mM [22]. In the case of samples with KCl, the collected CNF suspensions were acidified by adding 0.1 M HCl solution to pH 4.0, and KOH solution was added to reach pH 8. Then, KCl solution was added to control the ionic strength of the CNF suspension to 10 mM. To spray-dry CNF suspensions, an inlet temperature of 150 °C, gas flow rate of 540 L/h, pump rate of 4.5 mL/min, and drying gas flow rate of approximately 35 m^3^/h were used in the drying process. The outlet temperature of the spray-dryer was 89 °C. The dried CNFs were collected in plastic bags and stored in a desiccator at ambient temperature to prevent moisture absorption [6,15,23].

Then dried CNFs were redispersed by a Ultra-Turraxmixer (IKA T25, IKA-Werke GmbH & Co.KG, Staufen, Germany) in distilled water at 10,000 rpm for 30 s to reach a concentration of 1 wt% [17].

**Figure 1:**
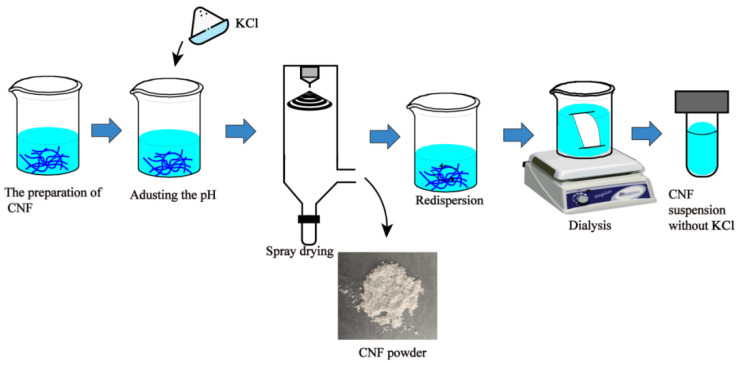
Procedure of preparing cellulose nanofibrils (CNF)-KCl-wash sample.

To investigate the effect of two salts on the redispersion of CNF during the redispersion process, five types of CNF samples were prepared. The never dried CNF was named CNF_0_, and CNF sample prepared by spray drying and resdispersion was named CNF. CNF sample with NaCl addition followed by spray drying and resdispersion was named as CNF-NaCl. CNF-NaCl-wash indicates the CNF sample redispersed with NaCl addition followed by spray drying, resdispersion and dialysis. For the CNFs with KCl addition followed by spray drying and resdispersion was named as CNF-KCl. After removing KCl from the CNF-KCl by dialysis, CNF-KCl-wash was obtained. For easy understanding, the procedure of preparing CNF-KCl-wash was illustrated in Figure 1.

### 2.4. Field-Emission Scanning Electron Microscopy (FE-SEM)

Field-emission scanning electron microscopy (FE-SEM) was conducted with an SEM (Hitachi Regulus^®^ Regulus 8220, Hitachi, Japan) working at a low acceleration voltage (5 kV) and short working distance (7.5 mm). Prior to imaging, the samples were coated with a 2 nm thick platinum/palladium in a Cressington sputter coater to ensure the conductivity [24].

### 2.5. Energy-Dispersive X-ray Spectroscopy (EDX)

To characterize the CNF and the salt added in the aqueous suspension, microscopy analyses were performed using a SEM coupled with an EDX (Octane pro silicon drift (SDD) detector, EDAX Inc., Mahwah, NJ, USA.), to track salt distribution on the sample surface [22].

### 2.6. Particle Size Distribution

A particle size analyzer (Mastersizer, ZS90, Malvern Instruments Ltd., Worcestershire, UK) was used to measure particle size of CNF redispersed in water at 25 °C. This technique allows the analysis of particles in the size range between 0.4 nm and 10 µm. A very dilute CNF suspension at 0.125 wt% of consistency was prepared by dispersing the sample with an ultrasonic homogenizer (Scientz-750 F, Ningbo Scientz Biotechnology Co, Ltd., Ningbo, China) for 30 s. Three measurements were made for each sample. The average of the measurements obtained from five samples was determined and reported [25].

### 2.7. Sedimentation Test

In order to measure the sedimentation degree of the samples, redispersed CNFs at 1 wt% were placed in vials and kept at room temperature for 60 min. The sedimentation degree of CNFs in the sample bottle was photographed with a camera.

### 2.8. Dispersion Stability Analysis

An optical analyzer (Turbisoft LAB Ageing Station, Formulaction Inc., Toulouse, France) was used to measure dispersion stability of samples. In this experiment, 1 wt% CNF suspension in a cylindrical glass cell was analyzed using a Turbiscan at 25 °C for 60 min. The stability of each sample was assessed based on the variation of transmission (ΔBS). The Turbiscan Stability Index (TSI) was calculated according to Equation (1):

(1) $$\mathrm{Turbiscan}\;\mathrm{Stability}\;\mathrm{Index}\;(\mathrm{TSI})\;=\;\sqrt{\frac{\sum_{i=1}^{n}\left(x_{i}-x_{\mathrm{BS}}\right)}{n-1}}$$

where x_i_ = average backscattering for three minute measurement; x_BS_ = Average value of x_i_; n = the number of scans [26]. In order to better evaluate the effect of salt on the dispersibility of redispersed CNF in aqueous suspension after spray drying, we define a new index, D, which is calculated by Equation (2):

(2) $$D=\frac{{\mathrm{TSI}}_{2}}{{\mathrm{TSI}}_{1}}\times 100\%$$

TSI_1_ represents the TSI value of the CNF sample without salt treatment, and TSI_2_ represents the TSI value of the CNF sample with salt treatment. Both TSI values at 60 min were used to obtain D in Equation (2).

## 3. Results

### 3.1. Scanning Electron Microscopy

**Figure 2:**
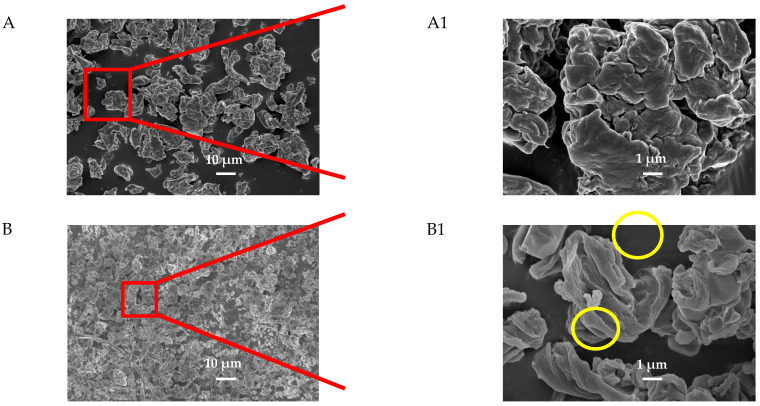

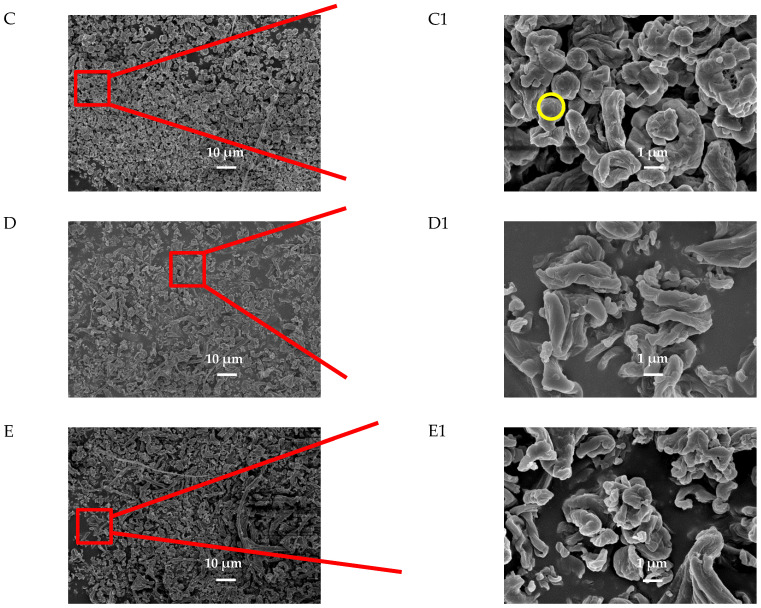
FE-SEM images of spray-dried CNFs samples (**A**) CNF, (**B**) CNF-NaCl, (**C**) CNF-KCl, (**D**) CNF-NaCl-wash, (**E**) CNF-KCl-wash. The images on the right side represents higher magnification. Yellow circle indicates the salt crystal.

To investigate the size of the spray-dried CNF samples, FE-SEM analyses were carried out. The morphologies of spray-dried CNFs were presented in Figure 2. It can be seen from Figure 2A that the CNF sample was large in size and showed serious acute agglomeration. CNF-NaCl was smaller in particle size and showed less agglomeration (Figure 2B) indicating that NaCl prevented the agglomeration of CNF during the spray drying process [18]. NaCl and KCl crystals were observed in CNF-NaCl and CNF-KCl. In particular, large granular NaCl crystals were present in CNF-NaCl (Figure 2(B1)), while less KCl crystals were observed in CNF-KCl (Figure 2(C1)). Interestingly, CNF-KCl was smaller and more homogenous compare to CNF-NaCl. This implied that CNF-KCl dispersed better or agglomerated less than CNF-NaCl in the spray drying process. Dialysis was so effective in desalination of the salts that no NaCl and KCl crystals were observed in CNF-NaCl-wash and CNF-KCl-wash samples (Figure 2D,E). The sizes of spray-dried CNF-NaCl-wash and CNF-KCl-wash were comparable to unwashed ones indicating the desalination did not cause any negative influence on CNF dispersion.

### 3.2. Energy-Dispersive X-ray Spectroscopy

The EDX device can follow the proportion of non-ionized elements with atomic numbers larger than 11. The relative amount of non-ionized salt in the CNFs is different depending upon the preparation conditions. “Non-complexation index” interactions between carboxylic groups and Na^+^ or K^+^ ions can be expressed as I_Na_/I_C_ or I_K_/I_C_ (I = intensity of the element) assumed to be similar to NaCl/CNF or KCl/CNF [22]. When the value is high, more Na^+^ or K^+^ atoms are detected, indicating that there is a higher amount of free Na^+^ or K^+^ ions.

Table 1 presents non-complexation indices (I_Na_/I_C_ or I_K_/I_C_) of the investigated CNFs, i.e., CNF-NaCl, CNF-KCl, CNF-NaCl-wash and CNF-KCl-wash. The lower the non-complexing index value, the better the complexing effect. It has been shown that CNFs may deprotonate and complex with Na^+^ [20,27]. As shown in Table 1, the I_Na_/I_C_ value of CNF-NaCl was 0.20 ± 0.01, while the I_K_/I_C_ of CNF-KCl was 0.15 ± 0.02. The I_K_/I_C_ value was lower than the I_Na_/I_C_, which indicated that there was more complexation between K^+^ and carboxyl groups in spray drying. This clearly showed that the hydrogen bond blocking effect of KCl during spray drying was stronger than NaCl. Compared with NaCl, KCl was more conducive to complexation.

### 3.3. Particle Size Distribution

**Figure 3:**
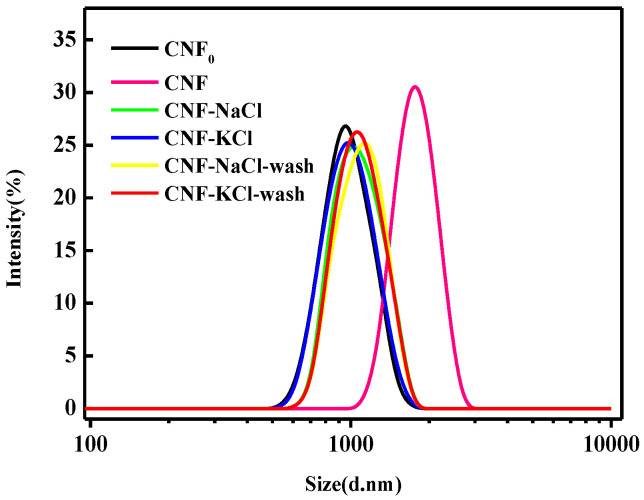
Particle size distributions of CNF_0_, CNF, CNF-NaCl, CNF-KCl, CNF-NaCl-wash and CNF-KCl-wash after redispersion in aqueous solution.

The particle size distributions of CNF_0_, CNF, CNF-NaCl, CNF-KCl, CNF-NaCl-wash and CNF-KCl-wash after spray drying were shown in Figure 3. It should be noted that the Malvern Mastersizer assumes the particles are spherical when calculating the particle size. This implies that the particle sizes and the size distribution data should be considered to be relative, because CNF has high aspect ratios (4–20 nm wide, 500–2000 nm in length) and deviates considerably from spherical geometry [28]. The mean size of CNF_0_ was 936 ± 11 nm, while the mean sizes of dried CNFs from CNF_0_, CNF, CNF-NaCl, CNF-KCl, CNF-NaCl-wash and CNF-KCl-wash were 1786 ± 15, 1045 ± 10, 1000 ± 8, 1091 ± 13 and 1033 ± 12, respectively. Since they were all dried by spray drying, the difference of the particle size might mainly be due to the difference of the atomized droplet sizes of CNF suspension during drying and the number of hydrogen bonds formed between CNF during drying. The particle sizes of the salt treated samples were significantly smaller than that of the untreated ones, which indicated that the salt treated CNFs agglomerated less during the drying process. In the samples treated with salt, the particle size of CNF-KCl was smaller than CNF-NaCl, which indicated that the degree of aggregation of CNF in CNF-KCl was smaller than CNF-NaCl. This was because K^+^ has a stronger charge absorption ability than Na^+^ [29,30] and shows a higher hydrogen bond blocking effect under the same drying conditions. In addition, the particle size of CNF-KCl-wash was smaller than CNF-NaCl-wash. We speculated that CNF-KCl-wash had a less tendency of agglomeration and hydrogen bond formation between CNFs.

### 3.4. Sedimentation of Redispersed CNFs

**Figure 4:**
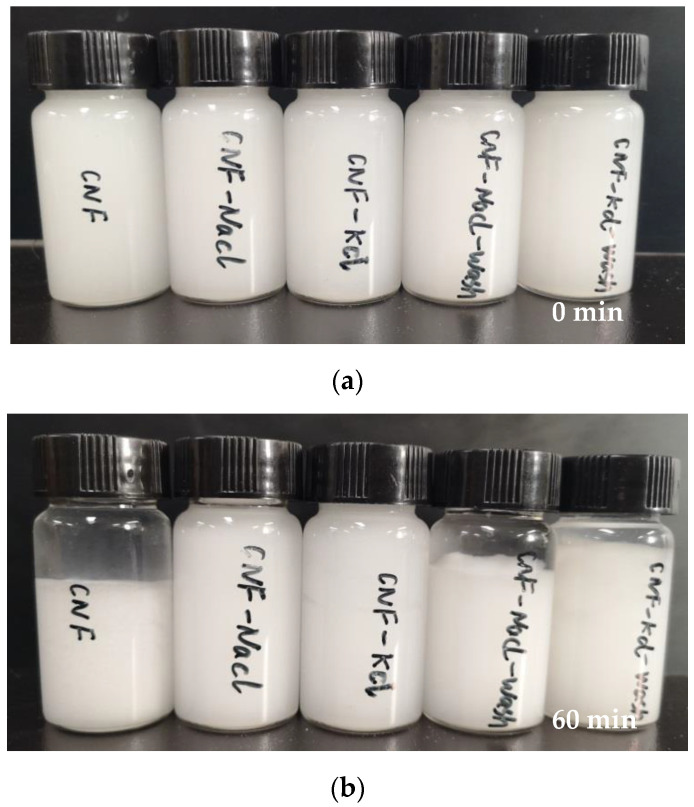
Images of sedimentation test after (**a**) 0 min and (**b**) 60 min.

The stability of the CNF dispersions in water was evaluated qualitatively by sedimentation tests. Figure 4b showed the CNF samples before and after the sedimentation experiment for 60 min. The consistency of all samples in Figure 4 was 1 wt%. CNF showed a clear boundary between the CNF sediment and supernatant. On the other hand, CNF-KCl was most well dispersed after 60 min of sedimentation, indicating its excellent redispersibility. The stability of the CNF dispersions was in the order of CNF-KCl, CNF-NaCl, CNF-KCl-wash and CNF-NaCl-wash.

This might be explained by that the added salt could be adsorbed on the surface of CNF to form an electric protective layer to prevent the coalescence between CNFs. The thermodynamic property of K+ was more active than that of Na+, and the formed electric protective film has stronger blocking effect on the coalescence between CNFs. From the viewpoint of dynamic stability, according to the equation of average displacement Δ when CNF does Brownian Motion Equation (3):

(3) $$\Delta =\sqrt{\frac{RTt}{L3\pi \eta r}}$$

where t was time; *T* was the thermodynamic temperature of the system; η was the viscosity of water; r was CNF radius; and L was Avogadro constant [31]. Because the dispersed *T* and η were the same, the average displacement of CNF particles was large and Brownian motion was obvious in the sample with small CNF radius within the same time, so as to counter the gravity effect and not accumulate and sink [31,32,33]. Therefore, the stability of the sample with smaller particle size was better, that is, the thickness of the upper clear layer was smaller. As shown in Figure 4b, after sedimentation for 60 min, the thickness of upper clear layer in CNF-KCl sample was the smallest, followed by CNF-NaCl, CNF-KCl-wash, CNF-NaCl-wash and CNF. This was also proved by the particle size distribution measurement in which CNF-KCl has the best redispersibility, followed by CNF-NaCl, CNF-KCl-wash, CNF-NaCl-wash and CNF.

### 3.5. Dispersion Stability Analysis

**Figure 5:**
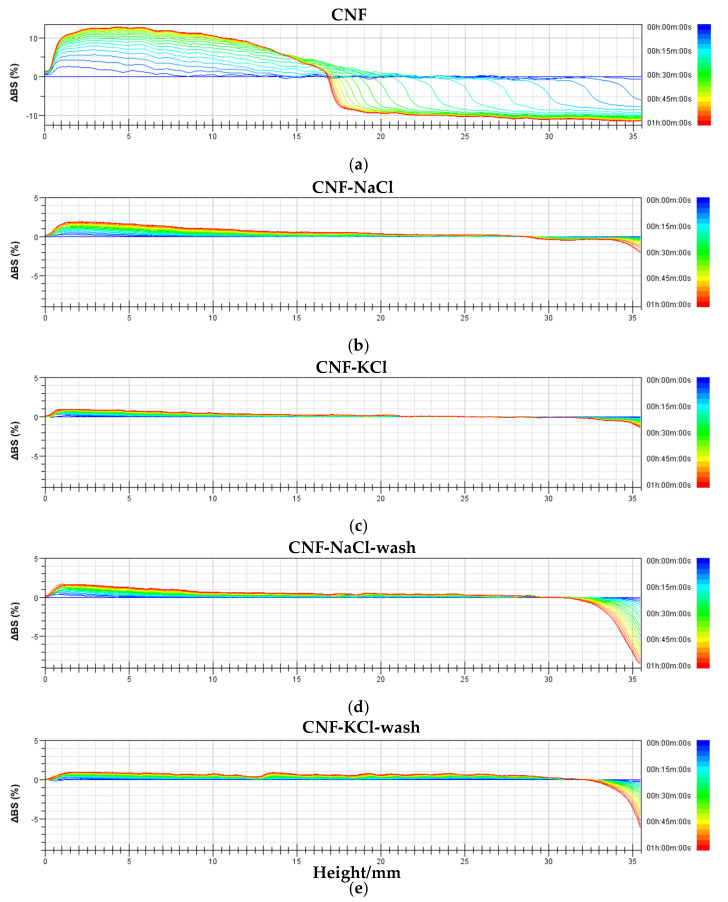
Effect of NaCl and KCl on the stability of (**a**) CNF, (**b**) CNF-NaCl, (**c**) CNF-KCl, (**d**) CNF-NaCl-wash and (**e**) CNF-KCl-wash (1 wt%) (measured by Turbiscan LAB Stability Analysis Tester within 60 min, the lines of different colors represent the changes in the light.

The destabilization kinetics of redispersed CNF suspensions was analyzed using TurbiSoft LAB. TSI of CNF aqueous suspension increased with time, which indicated that dispersed phase settled to the bottom of the vial because the concentration of CNF was lower than its gel point [8,34,35,36]. The Turbiscan ageing station spectra of the samples were shown in Figure 5. The stability of each sample was assessed based on the variation of transmission (ΔBS). Positive and negative values of ΔBS indicate the increase and decrease of CNF consistency, respectively, accompanied by the sedimentation process. Thus, as the sedimentation proceeded more CNF sedimented to the bottom of the test vial and caused an increase of ΔBS in the bottom [37]. From the backscattered light spectra in Figure 5, we could see that CNF has the largest degree of settling, the most obvious solid–liquid layering, the worst stability, and the fastest sedimentation speed, mainly because its particle size was the largest and therefore the worst dispersion in the sample. Due to the addition of salt, the particle size of CNF after drying was reduced while the stability of its aqueous solution was improved. The thickness of the upper clear layer of CNF-KCl was thinner than that of CNF-NaCl. In the case of CNF-KCl, some CNFs still remained in the upper layer, while the upper part of CNF-NaCl was clear indeed, indicating the K^+^ that is conducive to improving the dispersion of CNF. CNF-NaCl-wash and CNF-KCl-wash samples showed low dispersion stability, which was attributed to the loss of salts after dialysis that reduced the dispersion stability. However, the dispersibility of CNF-KCl-wash was better than that of CNF-NaCl-wash, which may be related to the smaller mean size of CNF-KCl-wash.

**Figure 6:**
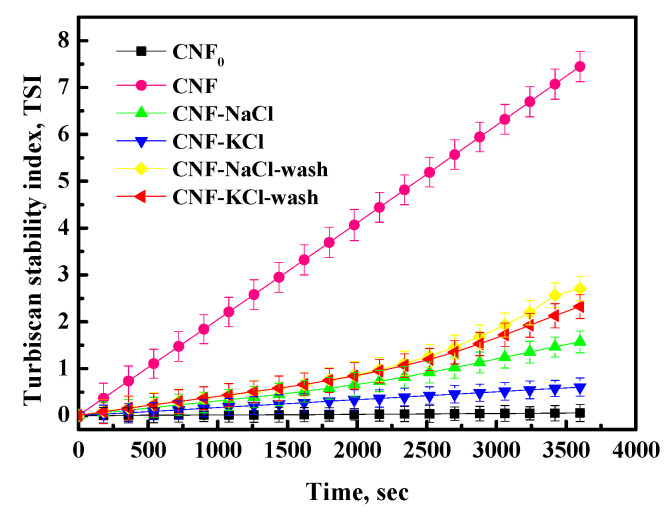
Turbiscan stability index of CNF samples.

The Turbiscan stability index (TSI) is a measure of the stability of the nanoemulsions. The better the stability of the sample, the smaller the TSI value. The TSI of all samples gradually increased with the time of sedimentation as shown in Figure 6. This might be explained that CNF suspension was a thermodynamically unstable system [32,33], CNF should subside as time increase until the CNF suspension reach a sedimentation equilibrium. It was observed that the TSI values of the salt samples CNF-NaCl and CNF-KCl were smaller than CNF-KCl-wash, CNF-NaCl-wash and CNF. This was due to the salt ions adsorbed on the surface of the CNF and form an electrical screening layer, which would change the agglomeration of the CNF thereby enhancing the stability of the suspension [21]. Compared with the TSI value of CNF-NaCl, the TSI value of CNF-KCl was lower. This may be due to K^+^ would form a stronger electrical protective film since K^+^ was more active than Na^+^ [29,30], which has a stronger blocking effect on the aggregation between CNF particles, thus improving the stability of the suspension, so its TSI value was lower than CNF-NaCl. From Figure 6, it could also be found that the TSI value of the dialysis sample was higher than that of the salted sample. This might be due to the loss of the electrical protection of salt and the stronger interaction between CNF. The TSI of CNF-KCl-wash was less than CNF-NaCl-wash, since particle size of CNF-KCl-wash was smaller than CNF-NaCl-wash, according to the Brownian Motion Equation (3), the smaller the particle size, the more stable the suspension was.

Table 2 showed the dispersion index of all samples. It could be seen that salt treatment would increase the water redispersibility of CNF particles after spray drying. However, the redispersibility of CNF reduced after dialysis. As shown in Table 2, the dispersion index of CNF-KCl was 8.06 ± 2.71, which was lower than that of CNF-NaCl (21.10 ± 3.27), and this implied that CNF-KCl had a higher redispersibility than CNF-NaCl. This was mainly due to the difference in the particle size of CNF in its suspension and the electrical protective film formed on CNF surface. The difference in dispersibility between CNF-NaCl-wash and CNF-KCl-wash was mainly due to the difference in CNF particle size.

In summary, we could find that under certain circumstances, adding an appropriate amount of salt was beneficial to improve the stability and dispersibility of CNF after spray drying, and the effect of KCl was better than that of NaCl.

### 3.6. Proposed Mechanism

**Figure 7:**
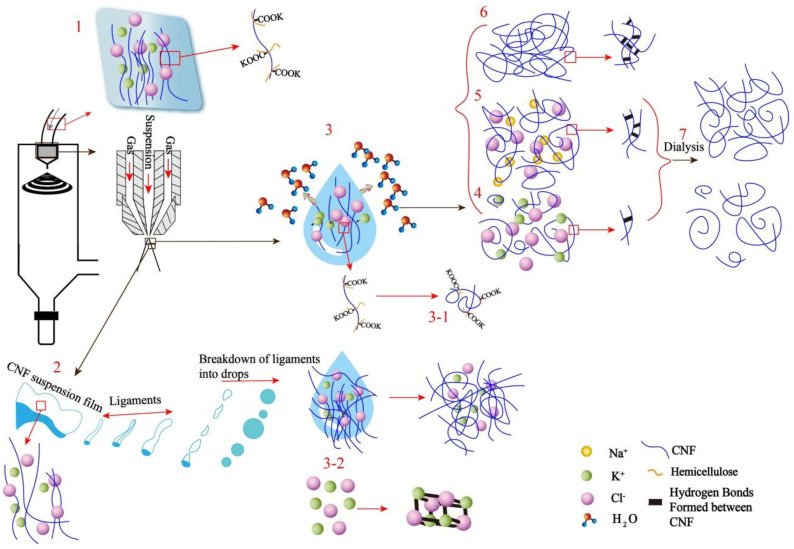
Schematic of proposed interaction between CNF and KCl (or NaCl) under spray drying.

Relatively low labor and maintenance costs indicate that spray drying could be used for those applications that require specific product characteristics. A schematic of the proposed interaction between CNFs and two monovalent salt (KCl and NaCl) under spray drying is shown in Figure 7. As shown in Figure 7-(1), before CNFs entered the dryer, the CNFs were a long strip with a high aspect ratio and had formed K-CNF (K-CNF was formed by carboxylate and potassium ion on hemicellulose). The CNF suspension was dried in a spray dryer (B-290, Switzerland) by atomization in contact with hot air, in which a two-fluid atomization system was used. In Figure 7-(2), the CNF suspension was first pumped through the nozzle and formed a suspension film. The flowing hot gas was used to mix the suspension externally. The momentum transfer between the gas and the suspension membrane breaks the suspension membrane into ligaments [38] and then turned into droplets with diameters from a few microns to tens of microns [39]. During this process there would be hydrogen bonds formation between the CNFs, but the addition of salt would block the formation of hydrogen bonds, making the suspension film easier to be split to form smaller diameter droplets. This was due to the fact that Na^+^ has a six-fold coordination which replaces the usual intramolecular hydrogen bond between O-3–H and O-5′ by a bridge involving Na^+^, O-3, O-5′, and O-6′ [40]. Since K^+^ has stronger charge absorption ability than Na^+^ [29,30], KCl has the stronger ability to block hydrogen bonding than NaCl. In addition, CNF may be atomized in two cases: CNF droplets without CNF fibrils protruding as shown in Figure 7-(3); part of CNF fibrils protruding as shown in Figure 7-(3-1) [6]. Due to the high temperature of the drying chamber, water droplets evaporated when passing through the drying chamber, and a large number of hydrogen bonds formed between CNF and clustered together to form spherical particles. On the other hand, in the second case, a portion of the original long CNF fibrils protruded outside the droplet. The corresponding drying mechanism was shown in Figure 7-(3-1). Since spray drying has no constant-speed drying period, agglomerated CNF particles were formed by attaching small CNF fibrils in suspension to longer CNF fibrils, and irregularly shaped particles were obtained. In this case, the principle of salt was the same, but the effect of blocking hydrogen bonds during drying was different. This was mainly because the force of KCl in CNF-KCl and the hydroxyl group on the CNF glucose unit was greater than in CNF-NaCl. It is worth mentioning that part of the salt added as shown in Figure 7-(3-2) may also recrystallize during the drying process. The morphology of CNF-KCl after drying was shown in number 4 of Figure 7: it was spherical and inevitable forms partial hydrogen bonds between CNFs, but it has the smallest amounts of hydrogen bonds among all samples, so its size was also the smallest of the samples. The morphology of CNF-NaCl after drying was shown in number 5 of Figure 7: it was also spherical, but a certain number of hydrogen bonds formed between CNFs during drying, which was larger than that of CNF-KCl, its size was also larger than CNF-KCl. The morphology of CNF after drying was shown in number 6 of Figure 7. Although it was spherical, it has the largest number of hydrogen bonds and the largest size among all samples because no hydrogen bond blocker was added. As shown in number 7 of Figure 7, the sample dialyzed after adding salt could be collected and reused by removing the salt using osmotic pressure.

## 4. Conclusions

CNFs adsorbed with two types of monovalent salts (NaCl or KCl) were spray-dried to obtain dried CNFs. The mean particle sizes of CNFs measured by particle size analyzer showed that the CNFs adsorbed with KCl were smaller than those of NaCl. EDX experiments indicated that K^+^ is easier to complex with carboxyl, hydroxyl groups favorable for CNF redispersion. Moreover, the TSI Turbiscan stability index (TSI) values of the CNFs adsorbed with KCl were lower than those of the NaCl treatment, which indicated that the stability of the redispersed CNFs by KCl treatment was better than CNFs treated by NaCl. The results illustrated that KCl treatment was preferred to limit the irreversible agglomeration of CNFs in the drying process, making CNF easy to implant into the traditional polymer industry. Furthermore, monovalent salts (KCl and NaCl) are relatively cheap, and the process does not require any organic solvents or hazardous chemicals. Therefore, drying with KCl may be considered as a green and economically facile method for production of highly redispersible dried CNFs for large-scale industrial applications of CNFs in composites industries.

## Figures and Tables

**Table 1:** Intensity value of the tested atom and complexation index measured by EDX.

Samples	I_O_	I_C_	I_Cl_	I_K_	I_Na_	I_Na_/I_C_	I_K_/I_C_
CNF	51.54 ± 0.21	48.47 ± 0.18	0	0	0	0	0
CNF-NaCl	30.21 ± 0.28	48.61 ± 0.13	11.76 ± 0.01	0	9.46 ± 0.02	0.20 ± 0.01	0
CNF-KCl	26.52 ± 0.31	58.29 ± 0.11	6.72 ± 0.02	8.47 ± 0.03	0	0	0.15 ± 0.02
CNF-NaCl-wash	52.44 ± 0.21	47.57 ± 0.10	0	0	0	0	0
CNF-KCl-wash	52.54 ± 0.30	47.46 ± 0.14	0	0	0	0	0

**Table 2:** Dispersion index of samples.

	CNF-NaCl	CNF-KCl	CNF-NaCl-Wash	CNF-KCl-Wash
D (%)	21.10 ± 3.27	8.06 ± 2.71	36.29 ± 3.67	31.18 ± 3.58

## Data Availability

The data presented in this study are available on request from the corresponding author.
